# The history of the emergence and transmission of human coronaviruses

**DOI:** 10.4102/ojvr.v88i1.1872

**Published:** 2021-02-10

**Authors:** Elijah N. Mulabbi, Robert Tweyongyere, Denis K. Byarugaba

**Affiliations:** 1 Department of Veterinary Medicine, Faculty of Veterinary Medicine, Animal Resources and Biosecurity, Makerere University, Kampala, Uganda

**Keywords:** *Coronaviridae*, host range, diversity and evolution, transmission dynamics, mutation, recombination

## Abstract

Human coronaviruses are known respiratory pathogens associated with a range of respiratory illnesses, and there are considerable morbidity and hospitalisation amongst immune-compromised individuals of all age groups. The emergence of a highly pathogenic human coronavirus in China in 2019 has confirmed the long-held opinion that these viruses are important emerging and re-emerging pathogens. In this review article, we trace the discovery and emergence of coronaviruses (CoVs) over time since they were first reported. The review article will enrich our understanding on the host range, diversity and evolution, transmission of human CoVs and the threat posed by these viruses circulating in animal populations but overtime have spilled over to humans because of the increased proximity between humans and animals.

## Introduction

Coronaviruses (CoVs) are positive-sense, single-stranded ribonucleic acid (RNA) viruses with a linear, non-segmented viral genome. Amongst all the RNA viruses, they have the largest genome of about 27–32 kb, packed in a helical nucleocapsid. They are named CoVs because of the crown-like appearance of the surface projections. Coronaviruses cause mainly respiratory and enteric diseases in mammals and birds with varying severity (Dijkman et al. 2013; Sipulwa et al. 2016; Weiss & Navas-Martin 2005). Coronaviruses are the largest group of viruses belonging to order Nidovirales, family *Coronaviridae*, in the sub-family *Coronavirinae*. The sub-family *Coronavirinae* is divided into four genera: the alpha (human coronavirus 229E [HCoV-229E] and human coronavirus NL63 [HCoV-NL63]); beta (human coronavirus OC43 [HCoV-OC43], human coronavirus HKU1 [HCoV-HKU1], severe acute respiratory syndrome coronavirus [SARS-CoV] and Middle East respiratory syndrome–related coronavirus [MERS-CoV]); gamma and deltacoronaviruses (Zhang et al. 2018). The gamma and deltacoronaviruses infect birds. Coronaviruses have a highly conserved genomic organisation with a single large 5’ open reading frame (ORF), constituting two-thirds of the genome encoding a replicase polyprotein, RNA-dependent RNA polymerase (RdRp) (Fehr & Perlman 2015). This is then followed by several additional ORFs that encode both structural and non-structural proteins. These structural proteins include the nucleocapsid (N) protein, matrix (M) protein, envelope (E) protein and the spike (S) protein (Masters & Perlman 2013). The spike protein is the major determinant of the virulence of CoVs because it mediates the binding of the virus to the specific host receptor.

## Host range

Coronaviruses exhibit a broad host range, infecting many mammalian and avian species, where they cause a broad spectrum of diseases in the respective hosts. The specificity to the host is because of the presence of specific receptors in the host, which interact with the CoV through the spike protein during attachment and entry into the host (Lim et al. 2016). It is by this convenience that most CoVs are named based on their host or site in the host at which the pathology manifests. Although this is so, there is a great possibility of cross infection of CoVs because of their ability to jump species barriers, which in result makes them have a broad host range (Cavanagh 2005). Some CoVs are known to be promiscuous being detected in more than one host, like some bovine coronavirus (BCoV, HCoV-OC43 and other related viruses), which have been detected in cows, humans, horses, dogs, camels, deer, antelopes and giraffes (Alekseev et al. 2008; Hasoksuz et al. 2007; Jin et al. 2007; Lim et al. 2013), whilst others like the most recently described deltacoronaviruses are host-specific and confined to a single host. Bats have been known to be reservoir hosts of alpha and betacoronaviruses, which predominantly affect mammals, whilst birds are reservoir hosts of gamma and deltacoronaviruses, which affect avian species and some mammalian species (Drexler, Corman & Drosten 2014; Woo et al. 2012). Specifically, the host of HCoV-229E are bats, HCoV-NL63 are bats and palm civets, HCoV-OC43 are cattle, HCoV-HKU1 are mice, SARS-CoV are bats and palm Civets, MERS-CoV are bats and camels, and for severe acute respiratory syndrome coronavirus 2 (SARS-CoV-2) the hosts are yet to be established but suspected to be bats and an unknown mammal intermediate host (Bolles, Donaldson & Baric 2011; Corman et al. 2018; Guo et al. 2020; Reusken et al. 2016). The host receptors of human CoVs have been identified: aminopeptidase N for HCoV-229E, angiotensin-converting enzyme 2 for SARS-CoV, SARS-CoV-2 and HCoV-NL63, CD26 commonly called dipeptidyl peptidase 4 for MERS-CoV and 9-O-acetylated sialic acid for HCoV-OC43 and HCoV-HKU1 (Bonavia et al. 2003; Hofmann et al. 2005; Huang et al. 2015; Lu, Hu et al. 2013; Van der Hoek 2007; Zou et al. 2020). Having noted earlier that CoVs have the ability to break species barriers, this definitively points to the variations that occur in the spike protein and ultimately the evolution of CoVs.

## The sequential discovery of human coronaviruses

Coronaviruses have been described for close to a century as avian infectious bronchitis virus (IBV), which is the first CoV to be isolated in 1937, followed by murine hepatitis virus (MHV), which was reported a decade later (Bailey et al. 1949; Cavanagh 2005; Cheever et al. 1949). This shows that animal CoVs have been known since the 1930s, including those that are highly pathogenic to animals, such as transmissible gastroenteritis virus (TGEV), bovine coronavirus (BCoV) and feline infectious peritonitis virus (FIPV) (Saif 2004). The first human CoV to be isolated was HCoV-229E during a study of respiratory illness amongst medical students in the University of Chicago and was named 229E because it was isolated from student specimen 229E (Hamre & Procknow 1966). Subsequently, in 1965, viruses B814 and HCoV-OC43 were isolated from patients with common cold. By inoculation onto organ cultures of the respiratory tract, these viruses together with HCoV-229E were shown to have similar morphological characteristics with the IBV of chicken that had been isolated earlier (Hamre & Procknow 1966; McIntosh et al. 1967; Tyrrell & Bynoe 1966). Since then, several human and animal CoVs have been studied and have been shown to have a characteristic virion structure by electron microscopy after negative staining. The first diagnostic assays were based on tissue cultures and serological surveillance by measuring the significant rise in antibody titer. HCoV-229E and HCoV-OC43 were the only strains followed up after 1965 up to 1990s because they were the only strains that could easily be cultured, the other strains (OC16, OC37, OC38, OC44 and OC48) including the first identified human CoV, B814, were lost because of the inability to culture then; thus, no further investigations could be done on them (Van der Hoek 2007). With increasing research and discovery of new scientific techniques, the number and the importance of CoVs have grown exponentially; there has been discovery of many novel CoVs in animals and humans. Before 2002, only HCoV 229E and HCoV OC43 were known to be circulating in human population causing common cold, and they were relatively considered as harmless because they caused mild illness. However, in 2002, a CoV causing severe acute respiratory illness was discovered and was named severe acute respiratory syndrome coronavirus (SARS-CoV). Severe acute respiratory syndrome coronavirus initially emerged in 2002–2003 in Guangdong province in South China and caused severe respiratory infection with high morbidity and mortality that had not been experienced with CoVs before. The disease was more pronounced in the elderly, infants and those with underlying conditions that make them immunocompromised (Tsang & Zhong 2003). Although SARS-CoV disappeared a year after its emergence (Feng & Gao 2007), new strains of CoVs have continuously been discovered, and indeed in 2003, two more CoVs were discovered – HCoV-NL63 and HCoV-HKU1 (Drexler et al. 2014; Van der Hoek et al. 2004; Woo et al. 2005). HCoV-NL63 was isolated in the Netherlands in 2003 from a child suffering from bronchiolitis and conjunctivitis, and this was not an isolated case as the virus was identified in clinical specimens from seven additional individuals who included infants and adults (Van der Hoek et al. 2004). In 2004, HKU1 was discovered in Hong Kong from a 71-year-old man with pneumonia who had just returned from Shenzhen, China (Woo et al. 2005). Ten years after the discovery of SARS-CoV, another human CoV that causes severe respiratory illness was discovered in 2012, initially named Human Coronavirus Erasmus Medical Center (HCoV-EMC), but later the international committee on the taxonomy of viruses named it Middle East Respiratory Syndrome (MERS) CoV (Chan et al. 2015; De Groot et al. 2013; Zaki et al. 2012). MERS-CoV was first identified in Saudi Arabia from a 60-year-old man with acute pneumonia, developed respiratory distress, renal failure and finally succumbed to the disease (Widagdo et al. 2017). MERS-CoV causes a series of highly pathogenic lower respiratory tract infections with a higher case fatality rate of 35% compared to 11% of SARS-CoV (Chan-Yeung & Xu 2003; Donnelly et al. 2019). However, unlike SARS which disappeared a year after its discovery, MERS has remained causing outbreaks in the Middle East, notably in Saudi Arabia (2014–2016) and in South Korea (2015) (Berry, Gamieldien & Fielding 2015; Fehr & Perlman 2015). Just 7 years after the discovery of MERS-CoV, in December 2019, a novel human CoV that is similar to SARS-CoV was discovered and isolated from patients in the Hubei Province, China, suffering from pneumonia, but has since spread to many countries across all continents (Zhu et al. 2020). This novel virus causes coronavirus disease (COVID-19), which has been declared a pandemic by the World Health Organisation (Summers et al. 2020). This novel CoV, initially designated as 2019-nCoV but now named SARS-CoV-2, forms the seventh CoV that infects humans (Zhu et al. 2020).

## Diversity and evolution of coronaviruses

In all the CoVs that have so far been reported, occurrence has been spontaneous and their source has been mysterious. Schools of thought have suggested recombination and the low fidelity of the RdRp as the main contributing factors that drive the evolution and diversity of the different CoVs with their hosts.

Recombination involves the exchange of genetic information between two non-segmented RNA genomes, contributing to the genetic stability of picoraviruses and CoVs where it has been demonstrated to occur (Lai et al. 1985). Homologous recombination occurs between two regions of high sequence similarity of a single strain of a CoV. Ribonucleic acid recombination in CoVs can occur almost anywhere on the genome, forming many recombinants with multiple crossover events. This shows that CoVs have a high frequency of recombination, which gives them an emergence and epidemiological advantage (Lai 1992a). The frequency of recombination in CoVs is so high that even in the absence of selection pressure, recombinants can be formed and can become the most dominant population amongst the viruses (Lai 1992b; Simon-Loriere & Holmes 2011). Non-homologous recombination occurs if a cell is infected by two strains of a given species of CoV, and exchange between genetically different RNA genomes can occur resulting in a progeny with the sequence derived from both the parent strains (Cavanagh 2005; Simon-Loriere & Holmes 2011). Although the probability of this non-homologous recombination amongst distantly related strains is small because of the need of sequence similarity, it is quite common in CoVs.

Coronaviruses have a mutation frequency just like other RNA viruses because of the error-prone RdRp, which lacks a proofreading and mismatch repair capability. Mismatch repair processes are not possible for genomes of most RNA viruses because they replicate and package single-stranded genomes. The only mechanism of correcting the mismatch would be a replicase-associated proofreading mechanism that the polymerase lacks (Elena & Sanjuán 2005; Steinhauer, Domingo & Holland 1992). This makes CoVs have high mutation rates, which can result in potentially adaptively useful genetic variation (Drake et al. 1998). All these events, together with re-assorting mutations, give CoVs a potential to generate novel viral phenotypes by expanding their repertoire of essential genes with many new genes (Forni et al. 2017). This plasticity of the genome provides CoVs a selective advantage and fast adaptability to usual and unusual natural hosts propelling them along the evolution path and may yield virus strains of unexpected virulence.

All human CoVs originate from bats; so, bats definitely play an important role as the gene source in the evolution of human CoVs. In the lineage of bat CoVs, they are known to have jumped to other bat species and other mammalian species including humans, with each interspecies jumping resulting in dichotomous evolution to give rise to alphacoronaviruses and betacoronaviruses (Woo et al. 2012). This jumping phenomenon is quite common in the lineage of CoVs although the mechanism by which they jump and switch to new hosts is not clearly understood. The great diversity of CoVs circulating in the bats has been attributed to their rich species diversity, high population densities and the ability to fly over long distances. The frequency and diversity of CoVs in bats have been globally detected in all continents with appreciable genetic similarity to human CoVs (Anthony et al. 2017; Li et al. 2005; Tong et al. 2009).

## Inter-host transmission of coronaviruses: What is known

As noted earlier that bats and birds are reservoir hosts of all the CoVs and act as the gene source in the evolution pathway of these viruses, transmission starts from them although the mechanism of zoonotic transmission from bats to humans is unclear. It has, however, been suggested that intermediate hosts such as carnivores or herbivores are involved in the transmission of these viruses (Cotten et al. 2013; Enserink 2013; Graham & Baric 2010).

Human coronavirus 229E (HCoV-229E) and Alpaca-CoV have been shown to have a close relationship with the diverse CoVs that exist in hipposiderid bats in Africa, which are the natural hosts of that lineage (Corman et al. 2015). Humans can come into contact with hipposiderid bats in their natural habitats, which can suggest direct transmission of HCoV-229E from bats rather than from alpaca, which do not share habitats with these bats. However, further analysis shows that CoVs that have close genomic similarity with HCoV-229E occur in camelids and suggests that HCoV-229E evolved towards the human genotype in camelids thereby identifying camelids as the zoonotic source of human infection. The host switching of 229E virus from bats to humans must have been facilitated by deletions in the spike gene of the bat-associated 229E viruses (Corman et al. 2018).

Human coronavirus OC43 (HCoV-OC43) is a betacoronavirus. Betacoronaviruses have strains circulating in many highly divergent mammalian hosts such as primates, lagomorphs, artiodactyls, perissodactyls, rodents and carnivores (Alekseev et al. 2008; Drexler et al. 2014; Erles et al. 2003; Guy et al. 2000; Hasoksuz et al. 2007; Lau et al. 2012; Lim et al. 2013; Majhdi, Minocha & Kapil 1997; Tsunemitsu et al. 1995; Woo et al. 2014). Bovine coronavirus is the best studied representative of these animal CoVs; the great diversity of Beta-CoVs in livestock suggests them to be the zoonotic sources of HCoV-OC43. Like other CoVs, mutations in the spike gene reflect the adaptation of HCoV-OC43 to the human host. HCoV-OC43, like other Beta-CoVs, have no ancestral link to bats but rather to rodents because of their close similarity to mouse hepatitis virus (MHV), thus believed to have speciated in rodents (Corman et al. 2018). The mechanism of transmission from rodents to bovine is not well documented but the close proximity between humans and bovine causes the spillover to humans.

Human coronavirus NL63 (HCoV-NL63) has been found to be related to CoVs of bats in the families *Vespertillionidae* and *Hipposideridae*, which points to the ancestry origin of HCoV-NL43 (Drexler et al. 2010; Gloza-Rausch et al. 2008; Pfefferle et al. 2009; Tao et al. 2017). The mechanism of transmission of HCoV-NL43 to humans is not documented with no zoonotic reservoir so far identified.

There is no viral sequences relating HCoV-HKU1 to other animal species, save the relationship it has with rodent-associated viruses (Wang et al. 2015; Woo et al. 2005). Just like HCoV-NL63, the mechanism of transmission of HCoV-HKU1 to humans is not documented with no zoonotic reservoir so far identified.

Considering the recent epidemics in humans with the spread of SARS-CoV, MERS-CoV and SARS-CoV-2, new insights have occurred in the transmission patterns of CoVs.

SARS-CoV was previously thought to have originated from wild animals, the civet cats, raccoon, dogs and ferret-badger (Guan et al. 2003) but cumulative phylogenetic studies have pointed to a bat origin of SARS-CoV. This is because there is no evidence for the circulation of SARS-CoV like viruses in palm civets in both the wild and breeding facilities (Wang et al. 2006). Therefore, these animals are only incidental hosts of SARS-CoV, and the live markets were probably sites where the interspecies transfer of the animal virus to human occurred (Weiss & Navas-Martin 2005). SARS-CoV has been reported to infect macaque monkeys and domestic cats but transmission from these domestic cats to man has not been demonstrated (Fouchier et al. 2003; Navas-Martin & Weiss 2003). However, its ability to infect other animals suggests that SARS-CoV could be having a natural wild reservoir from which future outbreaks can originate.

The first search for the reservoir of MERS-CoV focused on bats because of the genetic close relatedness of MERS-CoV to Tylonycteris bat CoV HKU4 and Pipistrellus bat CoV KHU5. However, molecular clock analysis suggested that these bat CoVs are unlikely to be the direct ancestor of MERS-CoV (Chan, Lau & Woo 2013). Molecular and serological surveys in dromedary camels from Oman, Canary Islands, Qatar and Saudi Arabia give evidence that these animals are the reservoir of MERS-CoV (Azhar et al. 2014; Haagmans et al. 2014; Raj et al. 2014; Reusken et al. 2013). Dromedary camels in Saudi Arabia harbour several viral genetic lineages including those that actually caused human outbreaks, and in addition, an infectious virus has been isolated from them pointing at their important role in the transmission of MERS-CoV (Sabir et al. 2016). The closeness of dromedary camels to humans results in continuous zoonotic transmission of the MERS-CoV to humans, thus explaining the cause of new infection in humans compared to SARS-CoV, where no new infections have been reported in humans since January 2004 because there is limited human–bat or human–intermediate host interactions (Baseler et al. 2016; Wang, Potter et al. 2005).

Severe acute respiratory syndrome coronavirus 2 (SARS-CoV-2), the newly discovered human CoV, has been linked to a zoonotic source with a spillover to humans at Huanan Seafood Wholesale Market in Wuhan, China. There were a number of non-aquatic birds and rabbits that were on sale in that market before the outbreak (Mackenzie & Smith 2020). Through next-generation sequencing, SARS-CoV has been showed to be closely related to two bat-derived SARS-like CoVs, bat-SLCoVZC45 and bat-SLCoVZXC21, which have been previously characterised from bats in China (Hu et al. 2018). This is consistent with the fact that bats are natural reservoirs of CoVs. Just like other human CoVs that have intermediate hosts, it is likely that SARS-CoV-2 has an unidentified intermediate animal host sold in the seafood market from which the virus spread to humans (Li, Zai et al. 2020; Liu et al. 2020). This is so because there is no proof that there was proximity between humans and bats that host the virus, because in December the bats are known to have been hibernating (Lu et al. 2020). The genomic sequences of SARS-CoV 2 from different patients have a 99.9% identity, which suggested that the virus originated from one source within a very short period and spread quite rapidly.

## Human-to-human transmission of coronaviruses

Once zoonotic viruses have succeeded in breaking the species barrier to infect humans, their success in the human population depends on its ability to acquire sustained human-to-human transmissibility. Human-to-human transmission of CoVs occurs directly or indirectly through airborne route or contact route. Direct transmission requires the physical contact between an infected person and a healthy person, followed by the transfer of the virus through touching, contact with body fluids and inhalation of respiratory secretions. Indirect transmission requires contact between a susceptible person with a contaminated surface and resultant transfer of the virus into entry points into the body like the respiratory pathway.

The four human CoVs that are endemic in human populations and cause self-limiting common cold (HCoV 229E, HKU1, NL63 and OC43) are droplet-transmitted; these virus-containing droplets are released to the environment when an infected person breathes, coughs, sneezes or talks. This efficient human-to-human transmission of these virus is sustainable because these viruses replicate mainly in the central and upper parts of the respiratory tract (Perlman & Netland 2009; Richard et al. 2017; Wege & Ter Meulen 1982). Sustainable human-to-human transmission is also dependent on the stability of the viruses in the environment. Large virus-containing droplets released from infected persons do not remain suspended in the air for long but rather move for a short distance and surfaces or mucosa of close contacts. Smaller droplets can remain suspended in air for long period of time and carried further from the infected person. HCoV-NL63 can survive for 7 days in aqueous solution and respiratory sections and remains infective at room temperature for long (Abdul-Rasool & Fielding 2010; Müller et al. 2008). HCoV-229E can survive and be detectable after 6 days at 20 °C and 50% relative humidity; this survival can be enhanced further at lower temperature and higher humidity (Ijaz et al. 1985). Literature about the stability in the environment of HCoV-KU1 and HCoV-OC43 is scanty but human-to-human transmission is the documented mode of transmission.

Human-to-human transmission of SARS-CoV and MERS-CoV occurs through modes like droplets, direct human-to-human transmission and through fomites. However, long-term and sustained human-to-human transmission has not been established in these zoonotic viruses. MERS-CoV has a lower human-to-human transmission than that of SARS-CoV (Lu, Liu et al. 2013). Human-to-human transmission of SARS-CoV occurs rapidly, which explained the rapid spread of SARS across 29 countries within only 6 months (Groneberg et al. 2003; Peiris, Yuen et al. 2003). Three instances of laboratory-acquired SARS-CoV infection were reported in Singapore, China and Taiwan, but in each of these cases, there were no sustained human-to-human transmission to cause a threat of a recurrent global outbreak but rather highlighted the potential risk when biosafety procedures in the laboratory are not adhered to (Enserink & Du 2004; Liang et al. 2004; Lim et al. 2004). Human-to-human transmissions amongst family members contributed only 13% – 21% of the MERS cases and 22% – 39% SARS cases. This shows that the nosocomial route is the main route of transmission of these viruses because substantial virus shedding occurs only after the onset of symptoms and that is when patients seek treatment in medical centres (Anderson et al. 2004; Baseler et al. 2016; Cowling et al. 2015; Peiris, Lai et al. 2003). Studies showed that 44% – 100% of the MERS-CoV-infected individuals during the outbreak were linked to hospitals where they got the infection, and a similar observation was made for some clusters of SARS patients (Chowell et al. 2015; Hunter et al. 2016). MERS-CoV remains stable and viable for 48 h at 20 °C and 40% relative humidity, whilst SARS-CoV for 5 days at 22 °C – 25 °C and 40% – 50% relative humidity. However, both lose viability rapidly at a higher temperature and humidity (Van Doremalen, Bushmaker & Munster 2013). This stability enables these viruses to be transmitted as aerosols; because SARS-CoV is more stable, it has a more sustained transmission than MERS-CoV through aerosols. Fomite transmission of these viruses is possible because of the stability of these viruses; they stay on inanimate surfaces long enough such that by the time humans touch the surfaces, they get infected (Lee & Wong 2015). This explains the infection of humans who were not in close proximity with index patients in the respective outbreaks. SARS-CoV was shown to be excreted in stool and remains infectious in sewerage for 2 days; therefore, broken sewerage systems can be a mechanism of spread of SARS-CoV (Wang, Li et al. 2005).

The newly identified SARS-CoV-2 has been suggested to be transmitted through droplet transmission and human-to-human transmission as evidenced with clusters of infected families and medical workers who have not had any exposure to animal markets where the infection started (Carlos et al. 2020; Chang et al. 2020; Li, Guan et al. 2020; Wang et al. 2020). Just like other HCoVs, nosocomial transmission has been implicated as an important mode of acquiring infection; actually one study suggested hospital transmission to be 41% amongst the patients (Wang et al. 2020). The stability of SARS-CoV-2 on contaminated surfaces and remaining viable for hours in aerosols or days on surfaces allow virus transmission through aerosols and fomites (Van Doremalen et al. 2020). SARS-CoV-2 has been detected in stool, suggesting that viral shading in stool is a potential route of transmission when persons get in contact with contaminated sewerage and also points to the possibility of fecal-oral transmission of the virus (Young et al. 2020). In comparison with SARS-CoV and MERS-CoV, where intestinal infections were observed at some later stages of infection, patients infected with SARS-CoV-2 may harbour the virus in the intestine in the early or later stages of infection. Detection of SARS-CoV in oral swabs, anal swabs and blood point to body fluids as an alternative mode of transmission through body fluids (Zhang et al. 2020). There are more studies being done, which will improve our understanding on the human-to-human transmission dynamics of SARS-CoV-2.

## Conclusion

Human CoVs are increasingly becoming important emerging pathogens from the previously known mild infections to severe acute respiratory infection with high fatality rates. Like other emerging pathogens, human CoVs pose a challenge to science and medicine because of the scantly information about them before they emerge from initially unknown zoonotic sources. With the increasing human population and climate change, people have increased proximity with animals, be either encroaching on their habitats or trade, and therefore zoonotic spillover of these viruses is continuously likely to occur. The biggest challenge here is that therapies and vaccination remedies have not been developed to match the emergence of these viruses, leaving treatment to be limited to non-specific supportive therapy. Since bats have been known to be an important reservoir of human viruses for several years, there should be continuous efforts to characterise the CoVs circulating in bat populations. There is a need to study the mechanisms through which these viruses find their way to the intermediate hosts because these intermediate hosts play an important role in linking bats to human populations. The possibility of direct transmission from bats to humans can also be investigated. Not only should these intermediate hosts be identified but also viral surveillance studies have to be expanded to wild and domestic animals such as bovine, rodents and carnivores in order to establish the important human CoVs that occur in these animals before they spill over to humans and cause pandemics. Mutations and recombination that occur in RNA viruses should be the contributory factor in the emergence of these viruses where they become able to utilise the human receptors that are required for successful infection and the progress of disease. Therefore, there is continuous need to monitor the effect of these mutations that occur in these viruses over a given period of time such that their emergence does not take the population by surprise and also precautionary measures should be taken early enough. With lack of therapies, avenues of animal-to-human transmissions must be blocked. This will require stringent regulatory mechanisms to control the trading of wild species in markets, creating buffer zones around habitats of wild animals and also an overall change in the cultural practices of communities. These can be enriched with the other avenues that are popularly used such as case isolation, quarantine and limiting the overall population mobility in this era where there is rapid expansion of transport networks. The One Health concept will always be a great approach in detecting, containing and eliminating public health risks from zoonotic pathogens.
